# Comparison of two different folic acid doses with methotrexate – a randomized controlled trial (FOLVARI Study)

**DOI:** 10.1186/s13075-015-0668-4

**Published:** 2015-06-11

**Authors:** Varun Dhir, Amit Sandhu, Jasbinder Kaur, Benzeeta Pinto, Phani Kumar, Prabhdeep Kaur, Nidhi Gupta, Ankita Sood, Aman Sharma, Shefali Sharma

**Affiliations:** Department of Internal Medicine, Postgraduate Institute of Medical Education and Research, Chandigarh, 160020 India; Department of Biochemistry, Government Medical College and Hospital, Sector 32, Chandigarh, India

## Abstract

**Introduction:**

There is reasonable evidence that folic acid 5–10 mg per week leads to reduction in methotrexate (MTX) toxicity in rheumatoid arthritis (RA). However, this is based on studies conducted with lower MTX dosage than used currently. It is unclear whether higher doses of folic acid may be better in reducing toxicity.

**Methods:**

This was a double-blind randomized controlled trial of 24 weeks duration. To be eligible, patients should have rheumatoid arthritis (1987 American College of Rheumatology criteria), be 18–75 years of age, not be on MTX and have active disease as defined by ‘Modified Disease Activity Score using three variables’ (DAS28(3)) > 3.2. MTX was started at 10 mg/week and escalated to 25 mg/week by 12 weeks. Folic acid was given at a dose of 10 mg (FA10) or 30 mg per week (FA30). Co-primary endpoints were incidence of toxicity (undesirable symptoms and laboratory abnormalities) and change in disease activity by 24 weeks. Intention-to-treat and per-protocol analyses were performed.

**Results:**

Among 100 patients enrolled, 51 and 49 were randomized to FA10 and FA30 respectively. By 24 weeks, there were 6 patient withdrawals in either group and mean(±SD) dose of MTX was 22.8 ± 4.4 and 21.4 ± 4.6 mg per week (*p* = 0.1). Frequency of patients with undesirable symptoms was non-significantly lower by 7.4 % (95 % confidence interval −27.4 to 12.7 %) in FA10 compared to FA30. There was also no difference in frequency of transaminitis (>Upper limit of normal (ULN)) (42.6, 45.7 %, *p* = 0.7) or transminitis as per primary endpoint (>2xULN) (10.6, 8.7 %, *p* = 1.0) or cytopenias (4.3, 4.3 %, *p* = 0.9). There was no difference in the primary end-point of occurrence of any adverse effect (symptom or laboratory) in FA10 and FA30 (46.8, 54.3 %, *p* = 0.5). At 24 weeks, DAS28(3) declined in both groups by a similar extent (−1.1 ± 1.0, −1.3 ± 1.0, *p* = 0.2) and ‘European League Against Rheumatism’ good or moderate response occurred in 56.9 and 67.4 % (*p* = 0.3).

**Conclusions:**

Even with the high doses of MTX used in current practice, there was no additional benefit (or harm) of a higher dose of folic acid (30 mg/week) over a usual dose (10 mg/week).

**Trial Registration:**

Clinicaltrials.gov NCT01583959 Registered 15 March 2012

## Introduction

Soon after the discovery of folic acid in 1941, its chemical analog and antifolate, aminopterin was developed and used in rheumatoid arthritis (RA) [1]. It was quickly supplanted by another analog, amethopterin, better known as methotrexate (MTX), first used in RA in 1964 [2]. However, MTX came into regular use only after pivotal controlled trials in the 1980s confirmed its efficacy and long-term studies found it to have the highest patient retention rate of all disease modifying anti-rheumatic drugs (DMARDs) [3–6]. In the last two decades, MTX has attained the status of gold standard and is the anchor drug for RA, being used in nearly 80 % of patients [7, 8]. However, withdrawals can occur in up to 30–50 % patients, mainly due to toxicity, thus limiting its use and necessitating expensive and toxic therapies [9, 10]. In a previous study, we found that patients with adverse effects had higher disease activity, probably due to under-dosing of MTX [11]. Thus, strategies to reduce toxicity and optimize the efficacy of MTX have received increasing attention in the past few years [12, 13].

One evidence-based strategy to reduce toxicity is supplementing with folic acid. There is reasonable proof from three controlled trials, and many meta-analyses that folic acid reduces the toxicity of MTX and the numbers of patients dropping out of treatment due to intolerance [14–19]. Indeed, most major contemporary guidelines currently recommend that folic acid supplementation is used with MTX [20–22]; however, its use remains controversial. First, there is controversy over the optimum dose of folic acid. Although most reviews and guidelines suggest a dose of 5–10 mg per week, this is based on insufficient evidence [22, 23]. Indeed, the only study that compared different doses of folic acid was done 20 years ago with MTX limited to 9–10 mg per week [15]. With the current use of higher MTX doses, such as 25 mg per week, it is unclear whether the dose of folic acid should be increased. Second, there is controversy over the effect on the efficacy of MTX. A large controlled trial did find prescription of slightly higher doses of MTX to achieve similar efficacy in the folic acid group [16]. In addition, some studies have suggested a negative effect on efficacy [24]. Thus, our study was planned to answer two questions. First, will a higher folic acid dose (30 mg per week) reduce the toxicity of MTX compared to a lower dose (10 mg per week) in RA? Second, will a higher folic acid dose lead to blunting of the efficacy of MTX compared to a lower dose?

## Methods

### Study design

This was a pragmatic double-blind parallel-group randomized controlled trial. This study was conducted in a single center and patients were enrolled between August 2012 and August 2013. This study protocol was approved by the Institutional Ethics Committee at the Postgraduate Institute of Medical Education and Research (PGIMER). The study was conducted according to current regulations in the International Conference on Harmonization guidelines and the principles of the Declaration of Helsinki. Written informed consent was obtained from all the patients included in this study. The trial, Folic acid in variable doses in rheumatoid arthritis (FOLVARI), is registered (Clinicaltrials.gov NCT01583959, date of registration 15 March 2012).

### Participants

Participants enrolled into the study were patients with RA, who were 18–75 years of age, fulfilled the American College of Rheumatology 1987 revised criteria, and had active disease (defined as modified disease activity score using three variables (DAS28(3)) >3.2) [25, 26]. We excluded patients who were currently receiving MTX or had received it in the last 8 weeks or were currently on folic acid supplements. Patients with contraindication(s) to methotrexate were also excluded. This included patients who were breast feeding or pregnant; had chronic liver or kidney disease; had cytopenia (below the lower limit of normal) or transaminitis (aminotransferases above the upper limit of normal); had active infection, including but not restricted to HIV, or hepatitis B or C, or active tuberculosis.

### Study treatments

All patients were started on oral MTX at a dose of 10 mg per week. The dose of MTX was escalated by 2.5 mg every 2 weeks, subject to the patient not achieving remission nor having toxicity (evaluated every 8 weeks), till a maximum dose of 25 mg per week was reached (see *Study procedures*). Patients were randomized in a ratio of 1:1 to receiving folic acid at a dose of 10 mg/week (FA10 group) or 30 mg/week (FA30 group) for 24 weeks. For this purpose, weekly packs consisting of six identical tablets were given to the patients, one tablet from a packet to be taken every day of the week, except the day when they took MTX. The tablets in these packs consisted of either two tablets of 5 mg folic acid (and four tablets of identical placebo) or six tablets of 5 mg folic acid. Thus, in the former group, patients could be taking the two tablets of folic acid on any of the days of the week. At 16 weeks, at the treating physician’s discretion, another DMARD (leflunomide, hydroxychloroquine, or sulfasalazine) could be started (in the case of poor response). In addition, participants could receive a total of three intramuscular injections of depot methyl-prednisolone acetate 80 mg during the course of the study at the treating physician’s discretion (excluding the last 8 weeks of the study period).

### Study outcomes

The primary outcome was the occurrence of MTX toxicity during 24 weeks. This included undesirable symptoms experienced by patients (evaluated using a questionnaire) and laboratory abnormalities (cytopenia or elevation of transaminases). Cytopenia was defined as a platelet count <100 × 10^6^/L or white blood counts <4 × 10^6^/L. Elevation of transaminases was defined as values more than twice the upper limit of normal (normal = 40 IU/L). The co-primary outcome was change in disease activity at 24 weeks. Secondary outcomes were change in the level of red blood cells (RBC) (and serum) folic acid at 24 weeks (from baseline) and change in the functional status of patients (assessed using the Indian health assessment questionnaire (HAQ)) [27]. Other outcomes assessed were change in serum matrix-metalloproteinase-3 (MMP-3) levels at 24 weeks (from baseline) and change in serum levels of TNFα, interleukin-6 and interleukin-10 (in some patients).

### Study procedures

At baseline and every visit (8 weekly), all patients were examined and joint counts performed by a single physician (VD), who remained blinded to their group. The DAS28(3) was calculated using the formula: (0.56*√ (tender joints) + 0.28*√ (swollen joints) + 0.70*Ln (erythrocyte sedimentation rate)) *1.08 + 0.16 [26].

At baseline patients underwent investigations including chest and hand radiography, complete blood counts, erythrocyte sedimentation rate (ESR), renal and liver function tests, rheumatoid factor and anti-citrullinated peptide assay (ACPA). MTX was started at a dose of 10 mg per week, and patients were instructed to increase the dose by 2.5 mg after every two doses (weeks) till their next visit. Thus, at the first follow up visit (8 weeks), most patients were on a dose of 17.5 mg per week, whereas by the second visit (16 weeks) most were on 25 mg per week. A decision to continue with escalation of MTX was made based on disease activity (remission - yes or no) and laboratory abnormalities (none or mild, moderate or severe). Further escalation of MTX was stopped in patients with remission (DAS28(3) ≤2.6) or moderate laboratory abnormalities (platelet count 90–100 × 10^6^/L, white blood count 3.5–4.0 × 10^6^/L, or transaminases more than twice the upper limit of normal). MTX was stopped completely in patients with severe laboratory abnormalities (platelet count <90 × 10^6^/L, white blood count <3.5 × 10^6^/L, or transaminases more than three times the upper limit of normal). Patients with moderate or severe laboratory abnormalities were called earlier at 2–4 weeks and re-evaluated. When abnormality was resolved, MTX, or its escalation, was restarted. A patient was suspected to have pulmonary toxicity in the event of a cough lasting longer than 2 weeks that was non-responsive to antibiotics and associated with breathlessness or any respiratory failure.

At every visit, patients completed a questionnaire that contained a list of undesirable symptoms that instructed them to “Tick symptoms which are new AND temporally related to the day on which you take methotrexate”. The list included symptoms of nausea and vomiting, dizziness, fatigue and malaise, uneasiness, anorexia, dysguesia, headache, skin rash and itching, diarrhea and oral ulcers. In addition, blood counts and transaminase levels were assessed at every visit. At baseline and 24 weeks patients were administered the Indian HAQ [27]. At every study visit a blood sample was collected in a citrate and plain vacutainer for ESR and serum separation (stored at −80 °C), respectively. RBC and serum folate levels were measured at baseline and 24 weeks, using chemiluminescent immunoassay on ADVIA Centaur (Siemens Healthcare Diagnostics Inc, IL, USA). To define low/deficient serum levels of folate, we used two cutoffs - 3 ng/ml (Institute of Medicine, NIH) and 4 ng/ml (World Health Organization (WHO) technical consultation) [28, 29]. Low RBC folate levels were defined using cutoffs of 140 ng/ml (Institute of Medicine, NIH) and 151 ng/ml (WHO technical consultation). Levels of MMP-3 were measured using ELISA (Human Total MMP-3 DuoSet, RnD). In a few patients, serum levels of TNFα and interleukin-6 were determined using cytokine bead array (BD Cytometric Bead Array (CBA), Becton Dickinson, La Jolla, CA, USA).

### Randomization and blinding

This was a double-blind study in which the participants, physician assessor and drug dispensing personnel were blinded to the dose of folic acid being administered. Initially, weekly packets consisting of six tablets - either two tablets of 5 mg folic acid (and four tablets of identical placebo) or six tablets of 5 mg folic acid were prepared and put into separate boxes. A colleague coded boxes as A or B, and kept the code secret till completion of the analysis. Randomization codes for individual patients (A or B) were generated using an online random number generator available on the website randomization.com (http://www.randomization.com, seed 20243) using permuted-block randomization (variable-sized blocks of 4, 6 and 8). Allocation concealment was maintained using serially numbered opaque envelopes and identical weekly packs and tablets.

### Statistical analysis

We assumed the frequency of toxicity (symptoms or laboratory) as 60 % in the 10-mg folic acid group, and estimated that it would halve to 30 % in the folic acid 30-mg group. Keeping the alpha error probability of 0.05 and power of 80 %, using a two-sided test we estimated the sample size required to be 48 in each group (G*Power 3.1) [30]. Comparison of continuous variables was done using the independent *t* test and categorical variables using the chi squared (*χ*2) or Fisher’s exact test, using SPSS (Version 20, SPSS Inc., Chicago, IL, USA). Both intention-to-treat and per-protocol analyses were performed. Graphical illustrations were prepared using GraphPad Prism (version 5.00, GraphPad Software, San Diego, CA, USA).

## Results

**Fig. 1:**
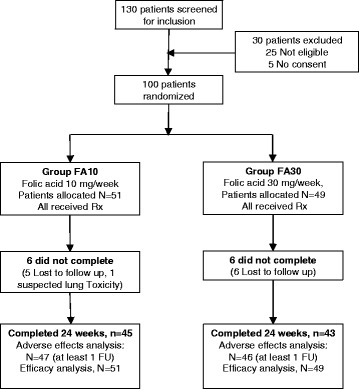
Flow chart showing flow of participants in the study. *Rx* treatment, *FU* follow up

A total of 130 patients with rheumatoid arthritis were assessed for eligibility, resulting in the inclusion of 100 patients (Fig. 1). Of these, 51 were randomized to receive folic acid at a dose of 10 mg per week (FA10), while 49 patients were randomized to 30 mg per week (FA30). These two groups did not have any significant differences in demographic and baseline characteristics (Table 1). By 24 weeks, there were six patient withdrawals in each group. Out of these, one patient (FA10 group) was withdrawn due to suspected pulmonary toxicity, and the remaining patients were lost to follow up. Mean (± SD) weekly dose of methotrexate at 24 weeks was not significantly different in FA10 and FA30 (22.8 ± 4.4, 21.4 ± 4.6 mg, *p* = 0.1). At 24 weeks, 38 and 29 patients in FA10 and FA30 were on an MTX dose of 25 mg per week (*p* = 0.1). The common reason for not reaching a dose of 25 mg/week was adverse effects (Table 2). After 16 weeks of MTX monotherapy, 29 patients had been started on an additional DMARD. Leflunomide was added in 28 patients (18 and 10 in FA10 and FA30, *p* = 0.1) and sulfasalazine in one patient (FA10 group).

**Table 1:** Baseline characteristics and demographics of patients included in the study

Mean ± SD (except where stated otherwise)	Folic acid 10 mg per week (FA10)	Folic acid 30 mg per week (FA30)	*P* value
	N = 51	N = 49	
Age, years	44.8 ± 10.0	43.8 ± 11.9	0.7
Female, number (%)	44 (86)	41 (84)	0.8
Duration of disease, years	4.4 ± 4.1	5.2 ± 5.1	0.4
Disease activity score (DAS28(3))	5.8 ± 0.9	5.7 ± 0.9	0.9
Tender joint count, 0–28	11.0 ± 4.8	11.2 ± 5.2	0.8
Swollen joint count, 0–28	5.1 ± 3.3	5.6 ± 3.6	0.5
HAQ, 0–3	1.1 ± 0.5	1.1 ± 0.5	0.7
Hemoglobin, g/dl	10.8 ± 1.7	10.9 ± 2.0	0.7
Serum albumin, g/dl	4.0 ± 0.5	3.9 ± 0.6	0.7
ESR, mm, first hour	65.4 ± 33.2	59.5 ± 30.0	0.3
Rheumatoid factor-positive, number (%)	42 (84)	35 (71)	0.2
ACPA-positive, number positive/number tested (%)	45/48 (94)	39/43 (91)	0.6

*DAS28(3)* modified disease activity score using three variables, *HAQ* health assessment questionnaire (Indian), *ACPA* anti-citrullinated protein antibody

**Table 2:** Reasons for patients within both groups not reaching a dose of 25 mg per week of methotrexate

	Folic acid 10 mg per week (FA10)	Folic acid 30 mg per week (FA30)	*P* value
Adverse effects	4	8	0.2
Achieved remission	2	4	0.2
Lost to follow up	5	6	0.7
No reason determined	2	2	1.0
Total	13	20	0.1

Results are presented as number of patients

### Primary outcome

There was no difference in the frequency of patients who had undesirable symptoms related to MTX in either of the groups. The most common symptoms were nausea, dizziness and uneasiness. The frequency of patients who experienced any undesirable symptom was 7.4 % lower (but not significantly: CI −27.4, 12.7) in FA10 compared to FA30. Similarly, the frequency of patients with nausea was 15.7 % lower (but not significantly: CI −33.9, 2.5) in FA10 (Table 3). There was no difference between groups in the frequency of patients who developed transaminitis or cytopenia. Even on analyzing only those patients who received 25 mg of MTX per week, there was no difference (data not shown). There was no difference between the groups in the composite endpoint of occurrence of any adverse effect (symptoms or laboratory measures), observed in 46.8 and 54.3 % (*p* = 0.5) of the FA10 and FA30 groups, respectively (Table 3).

**Table 3:** Number (percent) of patients in the two groups who experienced methotrexate toxicity at any time during the study

Adverse effects/toxicity	Folic acid 10 mg per week (FA10)	Folic acid 30 mg per week (FA30)	*P* value
	N = 47^a^	N = 46^a^	
Undesirable symptoms			
Any undesirable symptom	18 (38.3)	21 (45.7)	0.5
Nausea or vomiting	10 (21.3)	17 (37)	0.1
Dizziness	6 (12.8)	1 (2.2)	0.1
Uneasiness	4 (8.5)	3 (6.5)	1.0
Fatigue	2 (4.3)	3 (6.5)	0.7
Loss of appetite	2 (4.3)	3 (6.5)	0.7
Dysguesia	1 (2.1)	1 (2.2)	1.0
Headache	0 (0)	4 (8.7)	0.06
Oral ulcers	0 (0)	2 (4.4)	0.2
Laboratory abnormalities			
Transaminitis (more than ULN)	20 (42.6)	21 (45.7)	0.7
Transaminitis (more than 2 × ULN)	5 (10.6)	4 (8.7)	1.0
Cytopenia	2 (4.3)	2 (4.4)	0.9
Primary outcome^b^	22 (46.8)	25 (54.3)	0.5
Other adverse effects			
Suspected pulmonary toxicity	2 (4.3)	0 (0)	0.5
Herpes zoster	2 (4.3)	1 (2.2)	1.0

Results are presented as number (percent) of patients. ^a^Included only those patients who came for at least one follow up visit; ^b^any adverse symptom or cytopenia or transminases elevated more than twice the upper limit of normal (*ULN*)

**Fig. 2:**
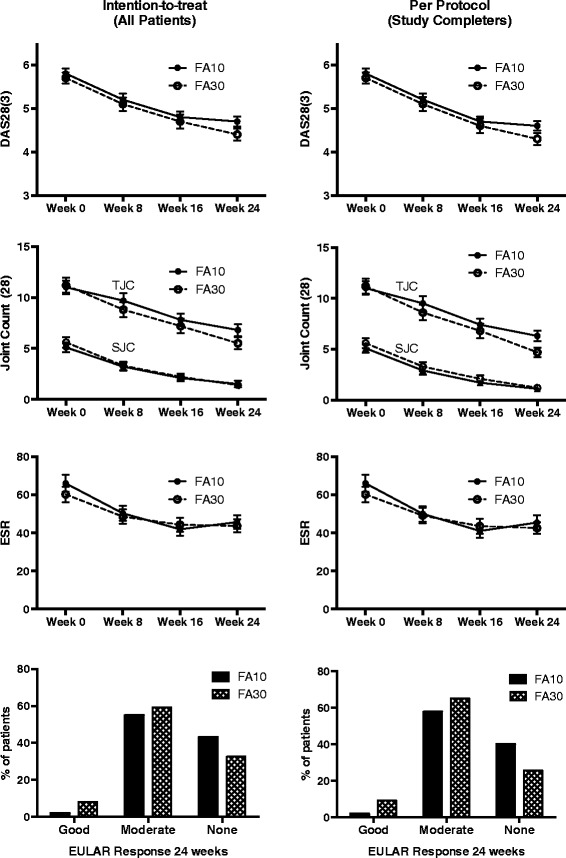
Change in disease activity (DAS 28(3)) and EULAR response criteria achieved by 24 weeks in the two groups. The *p* value was not significant at any time point. *DAS 28(3)* modified disease activity score using three variables, *EULAR* European League Against Rheumatism, *FA10* group in which folic acid 10 mg per week was given, *FA30* group in which folic acid 30 mg per week was given

There was no significant difference in mean (± SD) change in DAS28(3) in FA10 (−1.1 ± 1.0) and FA30 (−1.3 ± 1.0) (*p* = 0.2). At the 8-, 16- and 24-week time points, mean (± SD) DAS28(3) (and its components) was not significantly different between groups (Fig. 2). At 24 weeks, a good or moderate response according to the European League Against Rheumatism (EULAR) criteria occurred in 56.9 and 67.4 % patients in FA10 and FA30, respectively (*p* = 0.3). Even on per-protocol analysis, there was no difference in any of the efficacy measures (Fig. 2).

### Secondary outcomes

**Fig. 3:**
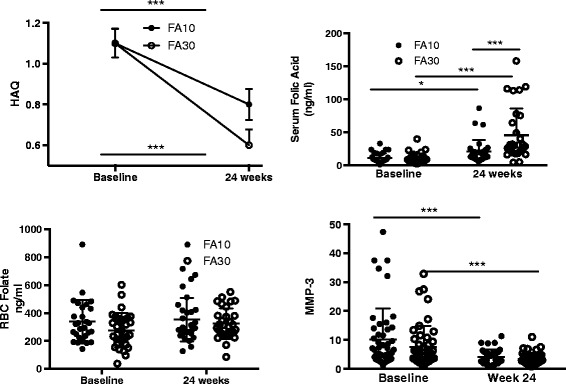
Change in mean Indian health assessment questionnaire (*HAQ*) scores, folic acid levels and matrix metalloproteinase-3 (*MMP-3*) in the two groups; *** *p* <0.001, *p <0.05. *FA10* group in which folic acid 10 mg per week was given, *FA39* group in which folic acid 10 mg per week was given

There was a significant increase in the serum folate levels in both groups from baseline to 24 weeks (measured in 68 patients); the level at 24 weeks in FA30 was almost twice that of FA10 (Fig. 3). However, there was no significant change in RBC folate levels in either of the groups. The HAQ score declined significantly and similarly in both groups (−0.3 ± 0.5, −0.4 ± 0.4, *p* = 0.27) (Fig. 3).

### Other outcomes

Pulmonary toxicity due to MTX was suspected in two patients (both in FA10), who had an unresponsive cough with dyspnea, one of whom one was restarted on MTX. Herpes zoster occurred in three patients during the study (Table 3). There was a significant decline in the serum MMP-3 levels at 24 weeks compared to baseline in both groups (*p* = 0.001), however, there was no significant inter-group difference at 24 weeks (Fig. 3). There was no significant change in the level of TNFα or interleukin-6 in any group and no difference between groups (measured in 15 patients, not shown).

### Folate levels at baseline

At baseline, low serum folate levels (in 95 patients in whom these were available) when defined using a value of <4 ng/ml were found in 9 patients (9.5 %) and in 4 patients (4.2 %) when defined using a value <3 ng/ml. Low RBC folate levels (in 85 patients in whom these were available), when defined using a value <151 ng/ml was found in 7 patients (8.2 %); 5 patients (6 %) were identified using a value of <140 ng/ml. Undesirable symptoms were significantly associated with low baseline serum levels of folate (for both cutoffs) (relative risk = 2.1 (CI 1.3–3.3)) but not with low RBC folate levels using either cutoff. However, there was no significant association between either low serum, or low RBC levels and the occurrence of transaminitis (>2 × ULN), cytopenia, or the composite primary endpoint. There was no correlation between the folate (serum or RBC) levels at baseline and the number of adverse effects (symptoms or laboratory measurements) (data not shown). Furthermore, even on excluding patients with low serum or RBC folate, there was no difference between groups in the primary endpoint.

## Discussion

Although, major guidelines and reviews recommend folic acid at a dose of 5 to 10 mg per week with MTX, to ameliorate toxicity, they acknowledge that ‘the evidence base is insufficient to determine the optimum dose’ and there may be ‘potential need for higher dosages, with the currently higher dosed methotrexate’ [22, 23]. Indeed, despite four randomized controlled trials on folic acid supplementation with MTX, only one has compared different doses of folic acid [14–17]. Even a recent meta-analysis on folic acid supplementation with MTX was restricted to the effect of low doses of folic acid (≤7 mg per week) [19].

This study, a randomized controlled trial, found no incremental benefit (further reduction of toxicity) with a higher dose of folic acid (30 mg per week) compared to the usual dose (10 mg per week). This was similar to the previous trial by Morgan et al*.* that compared 27.5 and 5.0 mg per week of folic acid [15]. However, there are important differences between these studies apart from the slightly different folic acid doses. The latter was conducted 20 years ago and the MTX dose was 9–10 mg per week compared to the mean dose of MTX, which was 21–22 mg in this study. That study used toxicity scores, whereas we have compared presence of symptoms, laboratory abnormalities and infections/lung toxicity separately. Although our study had small numbers, the confidence intervals suggest that higher-dose folic acid is unlikely to have a significant benefit in reducing either symptoms or laboratory abnormalities. However, it is difficult to interpret differences in the occurrence of uncommon toxicities such as pulmonary and herpes zoster that were higher, but not significantly so, in the 10-mg folic acid group.

Controversy remains on the effect of folic acid on the efficacy of MTX. Although no previous controlled trial has demonstrated this, the largest trial conducted showed a greater requirement for MTX in the folic acid group [16]. Another study, a post-hoc analysis of two different studies, found lower responses in the population supplemented with folic acid [24]. Also a study comparing different cohorts at different times periods found higher doses of MTX being used after folic acid fortification of food [31]. The latter two studies were inherently limited due to comparison of different populations (or across time). Our study cannot answer this question, as it lacked a placebo arm due to ethical considerations. However, our results suggest that any reduction in efficacy (if it occurs) does not have a dose–response effect. Indeed, we found a similar decline of DAS28(3) in both groups. This is further borne out by similar MTX doses in both groups and addition of a DMARD in similar numbers at 16 weeks. Additionally, decline in HAQ score, withdrawals in both arms and MMP-3 levels in both groups by 24 weeks were similar. Morgan et al. used discrete measures of activity, such as the joint index for tenderness or swelling and grip strength, and also found no difference between 5 mg and 27.5 mg folic acid per week [15].

We suggest that lack of any incremental benefit (i.e., higher folic acid leading to reduced toxicity) may be related inherently to the mechanism of action of folic acid. MTX has been shown to reduce intracellular folate levels in the liver, lymphocytes and RBCs [32, 33]. In addition, folate depletion is a risk factor for MTX toxicity [15]. Our results support the postulation that folic acid merely serves to correct the intracellular folate deficiency with MTX [15]. Indeed, the observed threshold of benefit of folic acid with MTX toxicity, can be explained by a dose of 5–10 mg per week to adequately correct this deficit. In addition, the lack of a dose–response effect of folic acid supplementation on efficacy does not support the alternate postulation, that folic acid reduces toxicity by directly antagonizing the action of MTX [34].

It is pertinent to mention differences with folinic acid. Many studies found high doses of folinic acid to affect MTX efficacy [35]. This may be related to two inherent differences from folic acid. One, folinic acid is a reduced and active folate that bypasses dihydrofolate reductase enzyme to provide the one-carbon folates necessary (unlike folic acid). Second, the transmembrane transport of folinic acid uses reduced folate carrier (RFC), that directly competes with MTX (folic acid uses a different channel - folate receptors) [36].

A limitation of our study is that some patients were started on another DMARD in the last 8 weeks (mainly leflunomide). This could have been responsible for some episodes of transaminitis and symptoms. However, we specifically asked patients to indicate adverse effects related to the timing of MTX use, and the results remained the same even after excluding the last 8 weeks. One of the strengths of this study is that it was done in a country (India) where there is no folic acid fortification program that may have confounded results in another country with such a program. We decided against including a group of patients on placebo (no folic acid supplementation) based on existing evidence at the time of planning the study. However, it would certainly have helped better clarify any effect on efficacy. The choice of dosage of folic acid in this study was partly related to logistics - only 5-mg tablets (and not 1-mg tablets) are marketed in our country. One valid criticism is that a dose of 5 mg once weekly (instead of twice weekly) may have been more appropriate as a comparator, as the former is more commonly used in many setups [22]. Also, in our study, patients in the 10-mg group could have taken their folic acid 5 mg tablets on any two days of the week except on the MTX day (randomly as they were given six identical tablets). Finally, our protocol of avoiding co-administration of folic acid on the same day as MTX, is not evidence-based, but has been suggested in certain guidelines and reviews [20, 23].

## Conclusions

We believe our study adds to the evidence on how best to supplement folic acid with MTX. Our results concur with the conclusions of many guidelines or reviews that 5–10 mg folic acid is enough, and higher doses do not offer any additional benefit even with the contemporary doses of MTX used in RA (such as 25 mg per week).

## Abbreviations

ACPA: anti-citrullinated protein antibody
DAS28(3): modified disease activity score using three variables
DMARD: disease modifying anti-rheumatic drug
ELISA: enzyme-linked immunosorbent assay
ESR: erythrocyte sedimentation rate
EULAR: European League Against Rheumatism
FA10: group in which folic acid 10 mg per week was given
FA30: group in which folic acid 30 mg per week was given
HAQ: health assessment questionnaire
MMP-3: matrix metalloproteinase 3
MTX: methotrexate
NIH: National Institutes of Health, USA
RA: rheumatoid arthritis
RBC: red blood cell
SJC: swollen joint count
TJC: tender joint count
TNFα: tumor necrosis factor alpha
ULN: upper limit of normal
WHO: World Health Organization

## References

[1] Gubner R, August S, Ginsberg V (1951). Therapeutic suppression of tissue reactivity. II. Effect of aminopterin in rheumatoid arthritis and psoriasis. Am J Med Sci.

[2] Black RL, O'Brien WM, Vanscott EJ, Auerbach R, Eisen AZ, Bunim JJ (1964). Methotrexate Therapy in Psoriatic Arthritis; Double-Blind Study on 21 Patients. JAMA..

[3] Weinblatt ME, Coblyn JS, Fox DA, Fraser PA, Holdsworth DE, Glass DN (1985). Efficacy of low-dose methotrexate in rheumatoid arthritis. N Engl J Med..

[4] Williams HJ, Willkens RF, Samuelson CO, Alarcon GS, Guttadauria M, Yarboro C (1985). Comparison of low-dose oral pulse methotrexate and placebo in the treatment of rheumatoid arthritis. A controlled clinical trial. Arthritis Rheum.

[5] Kremer JM, Lee JK (1988). A long-term prospective study of the use of methotrexate in rheumatoid arthritis. Update after a mean of fifty-three months. Arthritis Rheum.

[6] Weinblatt ME (2013). Methotrexate in rheumatoid arthritis: a quarter century of development. Trans Am Clin Climatol Assoc..

[7] Pincus T, Yazici Y, Sokka T, Aletaha D, Smolen JS (2003). Methotrexate as the "anchor drug" for the treatment of early rheumatoid arthritis. Clin Exp Rheumatol..

[8] Sokka T, Kautiainen H, Toloza S, Makinen H, Verstappen SM, Lund Hetland M (2007). QUEST-RA: quantitative clinical assessment of patients with rheumatoid arthritis seen in standard rheumatology care in 15 countries. Ann Rheum Dis..

[9] Alarcon GS, Tracy IC, Blackburn WD (1989). Methotrexate in rheumatoid arthritis. Toxic effects as the major factor in limiting long-term treatment. Arthritis Rheum.

[10] Rau R, Herborn G (2004). Benefit and risk of methotrexate treatment in rheumatoid arthritis. Clin Exp Rheumatol..

[11] Dhir V, Aggarwal A (2012). Methotrexate-related minor adverse effects in rheumatoid arthritis: more than a nuisance. J Clin Rheumatol..

[12] Kremer JM (2008). Methotrexate treatment of rheumatic diseases: can we do better?. Arthritis Rheum..

[13] Cipriani P, Ruscitti P, Carubbi F, Liakouli V, Giacomelli R (2014). Methotrexate in rheumatoid arthritis: optimizing therapy among different formulations. Current and emerging paradigms. Clin Ther.

[14] Morgan SL, Baggott JE, Vaughn WH, Young PK, Austin JV, Krumdieck CL (1990). The effect of folic acid supplementation on the toxicity of low-dose methotrexate in patients with rheumatoid arthritis. Arthritis Rheum..

[15] Morgan SL, Baggott JE, Vaughn WH, Austin JS, Veitch TA, Lee JY (1994). Supplementation with folic acid during methotrexate therapy for rheumatoid arthritis. A double-blind, placebo-controlled trial. Ann Intern Med.

[16] van Ede AE, Laan RF, Rood MJ, Huizinga TW, van de Laar MA, van Denderen CJ (2001). Effect of folic or folinic acid supplementation on the toxicity and efficacy of methotrexate in rheumatoid arthritis: a forty-eight week, multicenter, randomized, double-blind, placebo-controlled study. Arthritis Rheum..

[17] Griffith SM, Fisher J, Clarke S, Montgomery B, Jones PW, Saklatvala J (2000). Do patients with rheumatoid arthritis established on methotrexate and folic acid 5 mg daily need to continue folic acid supplements long term?. Rheumatology (Oxford)..

[18] Ortiz Z, Shea B, Suarez Almazor M, Moher D, Wells G, Tugwell P (1999). Folic acid and folinic acid for reducing side effects in patients receiving methotrexate for rheumatoid arthritis. Cochrane Database Syst Rev..

[19] Shea B, Swinden MV, Tanjong Ghogomu E, Ortiz Z, Katchamart W, Rader T (2013). Folic acid and folinic acid for reducing side effects in patients receiving methotrexate for rheumatoid arthritis. Cochrane Database Syst Rev..

[20] Chakravarty K, McDonald H, Pullar T, Taggart A, Chalmers R, Oliver S (2008). BSR/BHPR guideline for disease-modifying anti-rheumatic drug (DMARD) therapy in consultation with the British Association of Dermatologists. Rheumatology (Oxford)..

[21] Smolen JS, Landewe R, Breedveld FC, Buch M, Burmester G, Dougados M (2014). EULAR recommendations for the management of rheumatoid arthritis with synthetic and biological disease-modifying antirheumatic drugs: 2013 update. Ann Rheum Dis..

[22] Visser K, Katchamart W, Loza E, Martinez-Lopez JA, Salliot C, Trudeau J (2009). Multinational evidence-based recommendations for the use of methotrexate in rheumatic disorders with a focus on rheumatoid arthritis: integrating systematic literature research and expert opinion of a broad international panel of rheumatologists in the 3E Initiative. Ann Rheum Dis..

[23] Whittle SL, Hughes RA (2004). Folate supplementation and methotrexate treatment in rheumatoid arthritis: a review. Rheumatology (Oxford)..

[24] Khanna D, Park GS, Paulus HE, Simpson KM, Elashoff D, Cohen SB (2005). Reduction of the efficacy of methotrexate by the use of folic acid: post hoc analysis from two randomized controlled studies. Arthritis Rheum..

[25] Arnett FC, Edworthy SM, Bloch DA, McShane DJ, Fries JF, Cooper NS (1988). The American Rheumatism Association 1987 revised criteria for the classification of rheumatoid arthritis. Arthritis Rheum..

[26] Fransen J, van Riel PL (2005). The Disease Activity Score and the EULAR response criteria. Clin Exp Rheumatol..

[27] Kumar A, Malaviya AN, Pandhi A, Singh R (2002). Validation of an Indian version of the Health Assessment Questionnaire in patients with rheumatoid arthritis. Rheumatology..

[28] de Benoist B (2008). Conclusions of a WHO Technical Consultation on folate and vitamin B12 deficiencies. Food Nutr Bull..

[29] Institute of Medicine of the National Academies of Science (1998). Dietary reference intake for thiamine, riboflavin, niacin, vitamin B6, folate, vitamin B12, panthotenic acid, biotine and choline.

[30] Faul F, Erdfelder E, Lang AG, Buchner A (2007). G*Power 3: a flexible statistical power analysis program for the social, behavioral, and biomedical sciences. Behav Res Methods..

[31] Arabelovic S, Sam G, Dallal GE, Jacques PF, Selhub J, Rosenberg IH (2007). Preliminary evidence shows that folic acid fortification of the food supply is associated with higher methotrexate dosing in patients with rheumatoid arthritis. J Am Coll Nutr..

[32] Morgan SL, Baggott JE, Altz-Smith M (1987). Folate status of rheumatoid arthritis patients receiving long-term, low-dose methotrexate therapy. Arthritis Rheum..

[33] Kremer JM, Galivan J, Streckfuss A, Kamen B (1986). Methotrexate metabolism analysis in blood and liver of rheumatoid arthritis patients. Association with hepatic folate deficiency and formation of polyglutamates. Arthritis Rheum.

[34] Stenger AA, Houtman PM, Bruyn GA (1992). Does folate supplementation make sense in patients with rheumatoid arthritis treated with methotrexate?. Ann Rheum Dis..

[35] Joyce DA, Will RK, Hoffman DM, Laing B, Blackbourn SJ (1991). Exacerbation of rheumatoid arthritis in patients treated with methotrexate after administration of folinic acid. Ann Rheum Dis..

[36] Kremer JM (2004). Toward a better understanding of methotrexate. Arthritis Rheum..

